# Atypical Presentation of Spindle Cell Lipoma in a Young Male with a History of Malignant Melanoma

**DOI:** 10.3390/dermatopathology11040036

**Published:** 2024-11-26

**Authors:** Ty Theriot, John David Cattar, Lacey Falgout, Nicholas Culotta, Christopher Haas

**Affiliations:** Department of Dermatology, Louisiana State University Health Sciences Center, New Orleans, LA 70112, USAnculo1@lsuhsc.edu (N.C.); chaas2@lsuhsc.edu (C.H.)

**Keywords:** spindle cell lipoma (SCL), CD34, CD34-positive spindle cell tumors, myxoid spindle cell neoplasm, malignant melanoma, dermatofibrosarcoma protuberans (DFSP), solitary fibrous tumor (SFT), immunohistochemistry, atypical compound melanocytic nevus

## Abstract

Spindle cell lipoma (SCL) is a benign adipocytic tumor usually found in the subcutis of the posterior neck, upper back, and shoulder, predominantly in middle-aged males. This case report describes an atypical presentation of SCL in a 26-year-old male with a history of malignant melanoma. The patient presented with an erythematous plaque with central hyperpigmentation on the right upper arm, an uncommon location and presentation for SCL. Histopathological examination revealed an atypical myxoid spindle cell neoplasm with CD34 positivity and an overlying mildly atypical compound melanocytic nevus. The unusual clinical and histological features, combined with the patient’s melanoma history, complicated the differential diagnosis, which included dermatofibrosarcoma protuberans (DFSP) and solitary fibrous tumors (SFTs). A wide local excision with 2 cm margins was performed, and subsequent pathology confirmed clear margins, supporting the diagnosis of SCL. This case highlights the importance of including SCL in the differential diagnosis of CD34-positive spindle cell tumors, even when clinical and histological presentations are atypical, and underscores the need for thorough histopathological evaluation and a broad differential diagnosis in patients with a history of melanoma.

## 1. Introduction

Spindle cell lipoma (SCL), dermatofibrosarcoma protuberans (DFSP), and solitary fibrous tumors (SFTs) are cutaneous CD34+ spindle cell tumors that may exhibit histopathologic and immunophenotypic overlap [1]. SCL is a benign adipocytic tumor that primarily occurs in the subcutis of the posterior neck, upper back, and shoulder commonly appearing in middle-aged males. It presents clinically as a mobile, slow-growing, painless mass. Histologically, SCL has a variable distribution of mature adipocytes, bland spindle cells, collagen bundles., and pleomorphic/floret-like giant cells that are diffusely positive for CD34 on immunochemistry. Treatment often includes simple excision, and they display a low recurrence rate [2,3].

## 2. Case Report

**Figure 1:**
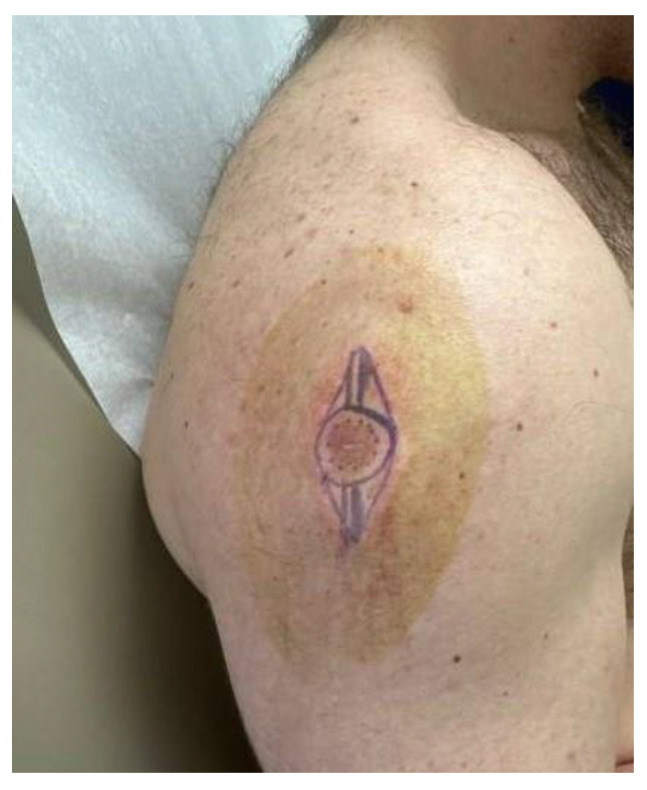
Clinical photograph of an erythematous plaque with central hyperpigmentation to the right upper arm in proximity to the shoulder.

**Figure 2:**
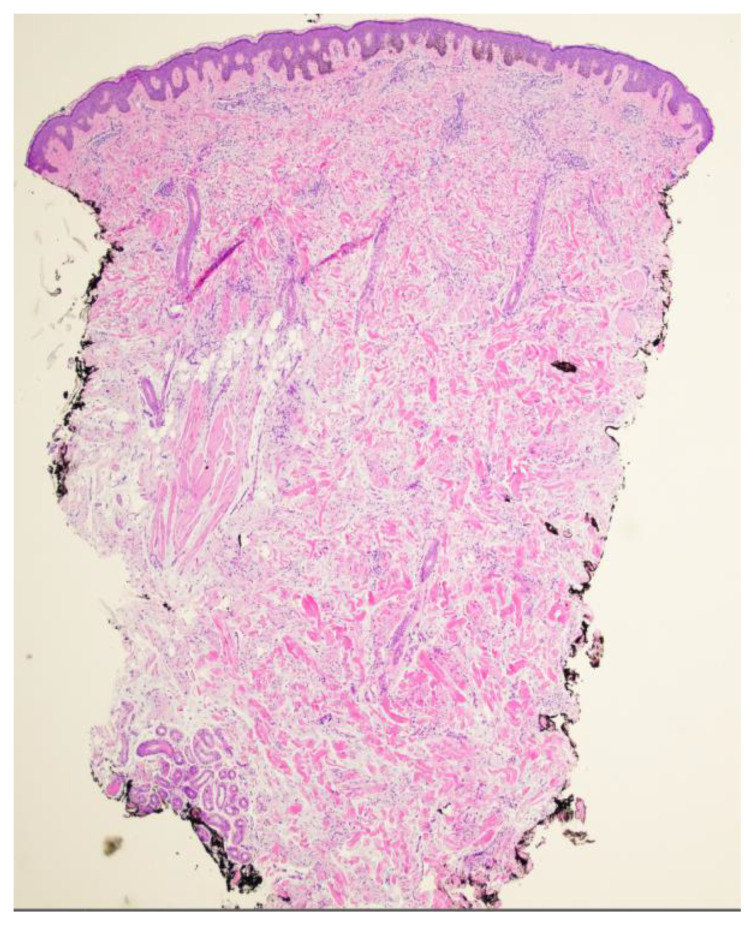
Punch biopsy at 2× magnification biopsy showing the proliferation of spindle cells in the dermis arranged in fascicles, mildly atypical melanocytic proliferation overlying.

**Figure 3:**
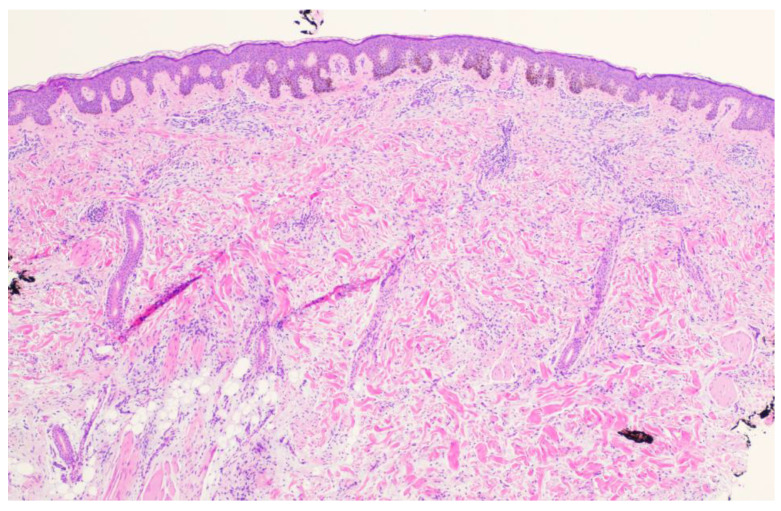
Punch biopsy at 4× magnification showing mildly atypical melanocytic proliferation and spindle cells in the dermis arranged in fascicles. A small amount of mucin is appreciable between the collagen bundles.

**Figure 4:**
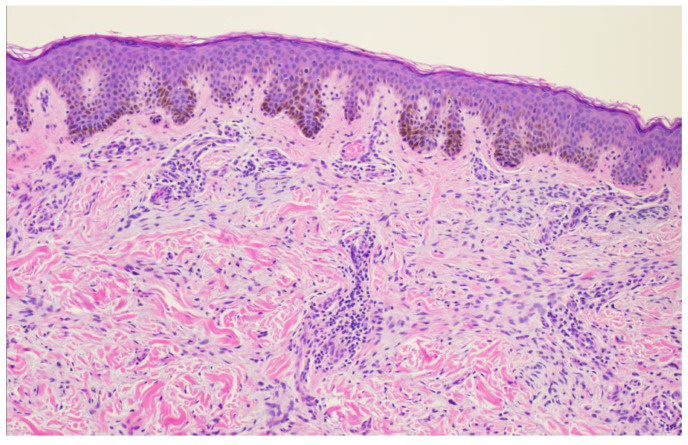
Punch biopsy at 10× magnification showing interface between mildly atypical nevus and spindle cell proliferation.

**Figure 5:**
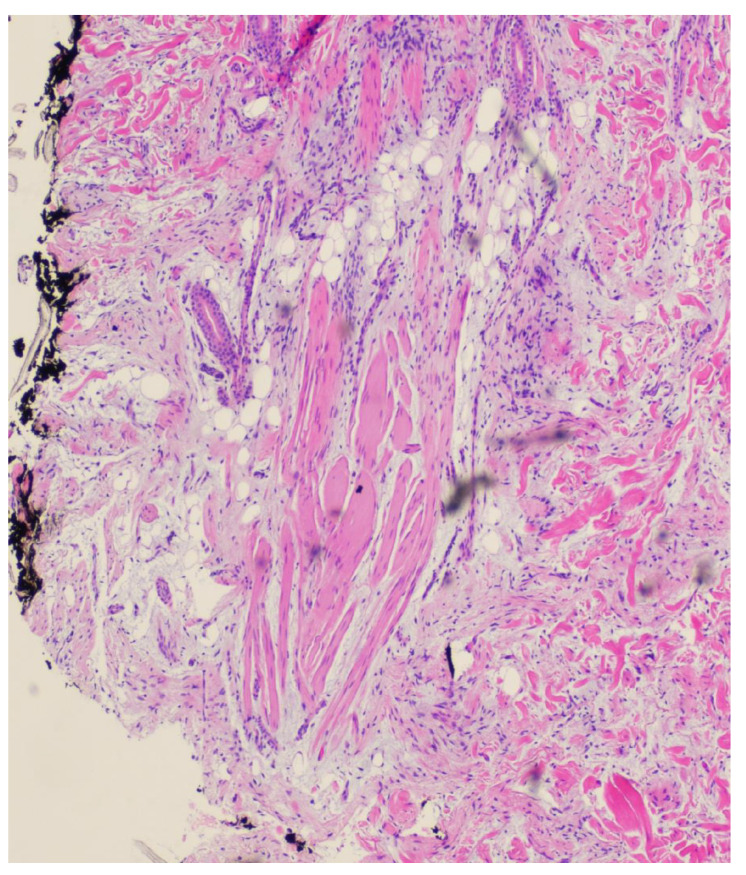
Punch biopsy at 10× magnification showing spindle cell proliferation surrounding adipocytes and adnexa.

**Figure 6:**
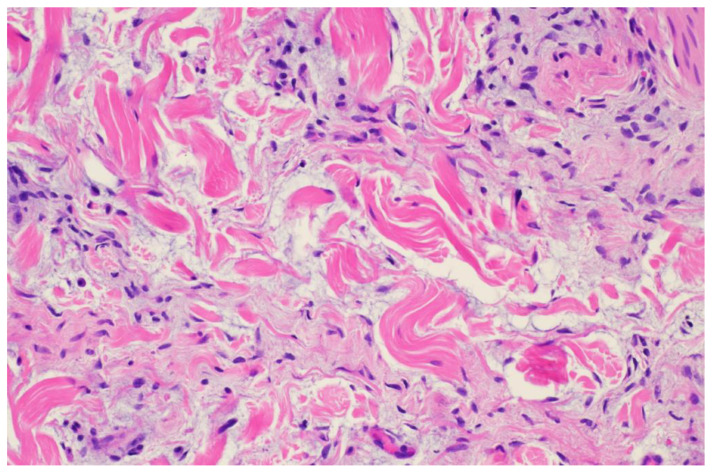
Punch biopsy at 20× magnification showing spindle cell proliferation with mucin deposition.

**Figure 7:**
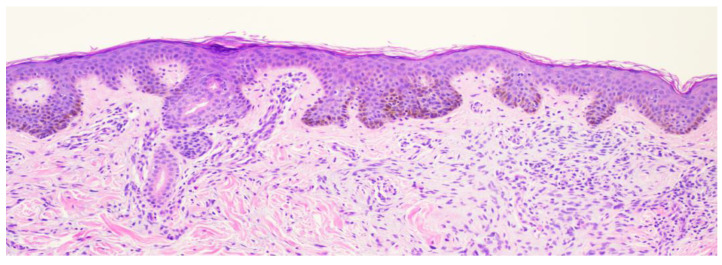
Punch biopsy at 10× magnification showing slight acanthosis with mild elongation of the rete ridges with melanocytic nests present at the dermoepidermal junction. Some bridging is present. Some melanocytes are noted in the superficial dermis.

**Figure 8:**
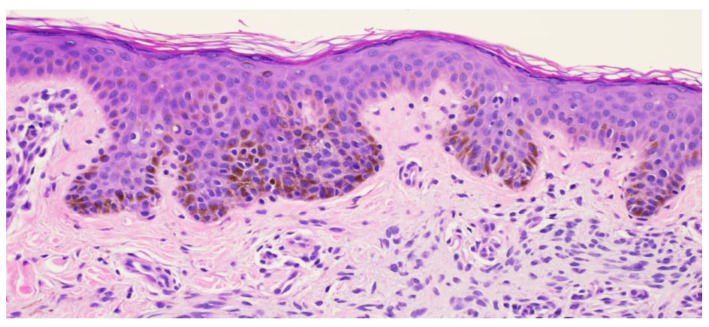
Punch biopsy at 20× magnification showing slight acanthosis with a mild elongation of the rete ridges. Melanocytes are present within the epidermis with small nuclei. Some melanocytes are noted in the superficial dermis.

**Figure 9:**
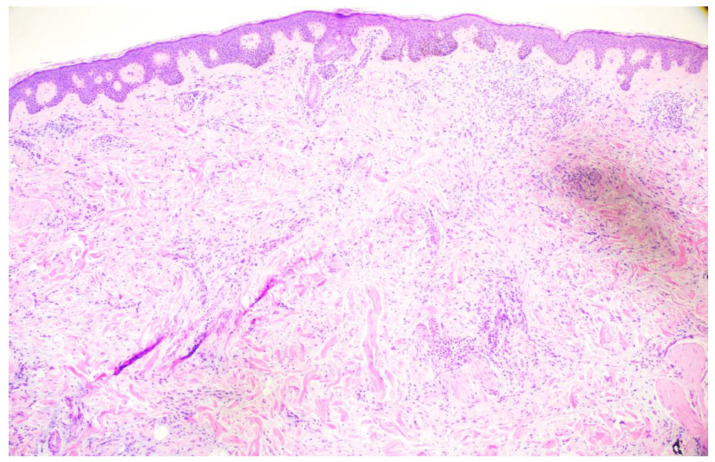
Punch biopsy at 4× magnification showing slight acanthosis with a mild elongation of the rete ridges. Some bridging is present. Few melanocytes are noted in the superficial dermis. Symmetric architecture is present.

A 26-year-old male with a history of 0.4 mm malignant melanoma of the left upper extremity status post wide local excision and tenuous sun protection presented to clinic for follow-up. Exam revealed multiple scattered nevi to the head, trunk, extremities, feet, and genitalia as well as an erythematous plaque with central hyperpigmentation to the right upper arm in proximity to the shoulder (Figure 1). A punch biopsy was obtained and revealed atypical myxoid spindle cell neoplasm and involved margins with overlying mildly atypical compound melanocytic nevus. The specimen also displayed CD34 positivity (histopathology displayed in Figure 2, Figure 3, Figure 4, Figure 5, Figure 6, Figure 7, Figure 8 and Figure 9). A diagnosis of spindle cell lipoma was reached, and wide local excision with 2 cm margins was performed and subsequent pathology revealed clear margins.

## 3. Discussion

This case highlights an atypical presentation of spindle cell lipoma. The patient was a 26-year-old male with a history of malignant melanoma, who presented with an erythematous plaque on the right upper arm near the shoulder, an area that is not typically associated with SCL. Furthermore, the clinical presentation included central hyperpigmentation, which is not a common feature of SCL. The initial differential diagnosis was broad due to the atypical appearance of the lesion and CD34 positivity.

Histopathologically, the presence of an atypical myxoid spindle cell neoplasm with CD34 positivity further complicated the diagnosis. The biopsy also revealed an overlying mildly atypical compound melanocytic nevus, which is an unusual finding in SCL and raised concerns about a potential collision tumor or a misdiagnosis.

Given the patient’s history of melanoma and the atypical features of the lesion, the decision to proceed with a wide local excision with 2 cm margins was prudent. The clear margins achieved on subsequent pathology confirmed the complete removal of the lesion, supporting the diagnosis of SCL despite its atypical presentation.

This case underscores the importance of considering SCL in the differential diagnosis of CD34-positive spindle cell tumors, even when the clinical and histological presentation deviates from the classic description. The unusual location, atypical histopathological findings, and the patient’s history of melanoma all contributed to the complexity of this case. The most unusual aspect of the case, and the reason it is being reported, is its superficial location, presentation in a young patient, and clinical morphology that ultimately was a spindle cell lipoma. It highlights the need for a comprehensive approach to diagnosis and management, including thorough histopathological evaluation and the consideration of a broader range of differential diagnoses when encountering unusual presentations of typically benign lesions.

## Data Availability

All referenced information can be found publicly in PubMed.
